# Path Smoothing Techniques in Robot Navigation: State-of-the-Art, Current and Future Challenges

**DOI:** 10.3390/s18093170

**Published:** 2018-09-19

**Authors:** Abhijeet Ravankar, Ankit A. Ravankar, Yukinori Kobayashi, Yohei Hoshino, Chao-Chung Peng

**Affiliations:** 1 School of Regional Innovation and Social Design Engineering, Faculty of Engineering, Kitami Institute of Technology, Kitami, Hokkaido 090-8507, Japan; hoshinoy@mail.kitami-it.ac.jp; 2 Division of Human Mechanical Systems and Design, Faculty of Engineering, Hokkaido University, Sapporo, Hokkaido 060-8628, Japan; ankit@eng.hokudai.ac.jp (A.A.R.); kobay@eng.hokudai.ac.jp (Y.K.); 3 Department of Aeronautics and Astronautics, National Cheng Kung University, Tainan 701, Taiwan; ccpeng@mail.ncku.edu.tw

**Keywords:** robot trajectory smoothing, robot navigation, path planning, autonomous vehicle motion planning

## Abstract

Robot navigation is an indispensable component of any mobile service robot. Many path planning algorithms generate a path which has many sharp or angular turns. Such paths are not fit for mobile robot as it has to slow down at these sharp turns. These robots could be carrying delicate, dangerous, or precious items and executing these sharp turns may not be feasible kinematically. On the contrary, smooth trajectories are often desired for robot motion and must be generated while considering the static and dynamic obstacles and other constraints like feasible curvature, robot and lane dimensions, and speed. The aim of this paper is to succinctly summarize and review the path smoothing techniques in robot navigation and discuss the challenges and future trends. Both autonomous mobile robots and autonomous vehicles (outdoor robots or self-driving cars) are discussed. The state-of-the-art algorithms are broadly classified into different categories and each approach is introduced briefly with necessary background, merits, and drawbacks. Finally, the paper discusses the current and future challenges in optimal trajectory generation and smoothing research.

## 1. Introduction

The advent of ubiquitous sensors [1], artificial intelligence, and decrease in the cost of computing [2] has set the stage for an increase in the number of service mobile robots [3]. Today, these robot are used for cleaning, industry automation, and moving stuff in the warehouse [4]. Service robots are also perfect for tasks which are dull, dirty, dangerous, and difficult. Most of these robots have the ability to navigate autonomously in an environment. For large environments and work on a large scale, multiple mobile robots are often used. These robots autonomously move between various locations of the environment to provide their services (like deliver items, or clean).

In order to navigate between different parts of the environment, mobile robots need a map of the environment. They also need to localize themselves in the map to estimate their current position. To do this, mobile service robots are generally equipped with exteroceptive sensors like monocular and stereo cameras, laser range finders, inertial measurement units (IMUs), and RGBD (RGB color information with depth) sensors. A SLAM (Simultaneous Localization and Mapping) [5,6] module builds the map of the environment and simultaneously localizes the robot in the map. The localized position of the robot is the start location, and the goal location is specified for the robot for it to navigate towards the goal using any of the state-of-the-art path planning algorithms. SLAM is considered to be a core module for autonomous mobile robots [7] because it is often a prerequisite to path planning, navigation, and manipulation for single and multi-robot systems [8,9,10,11].

In order to navigate towards its goal in a given map, a robot first requires to find the overall path from the start to the goal location. This involves considering the static obstacles of the map. The dynamic obstacles in the map (like moving people, other robots) are not considered at this stage. Various algorithms for path planning of autonomous robots have been proposed for planning this overall path. Each algorithm has its own advantages and disadvantages and a review of these algorithms can be found in [12,13,14]. For example, Dijkstra’s algorithm [15] with uniform cost search has widely been used for global planning. However, it is computationally expensive with a time complexity of $O(N^{2})$, where *N* is the number of nodes. Optimization of the Dijkstra’s algorithm has been proposed in [16] and variants like [17] have been proposed. Using a good initial heuristic, A-star algorithm [18] performs better than Dijksktra’s algorithm. On the other hand, D-star (or Dynamic A*) algorithm [19,20] finds an optimal path in real-time by incrementally updating paths to the robot’s state as new information is discovered, and is more efficient than A* algorithm. Rapidly exploring random tree (RRT) algorithm [21,22,23] efficiently searches high dimensional spaces by building space-filling trees randomly. It plans a path with a biased growth towards the goal with high probability. Other widely used path planning algorithms include potential fields [24] algorithm, and probabilistic road map (PRM) [25] planner. During the traversal of this overall path a robot might encounter dynamic obstacles like moving people or other robots and must change its trajectory accordingly. The dynamic obstacles are considered using various approaches like the “dynamic window approach” proposed in [26], and a smooth path is generated from the global path.

**Figure 1:**
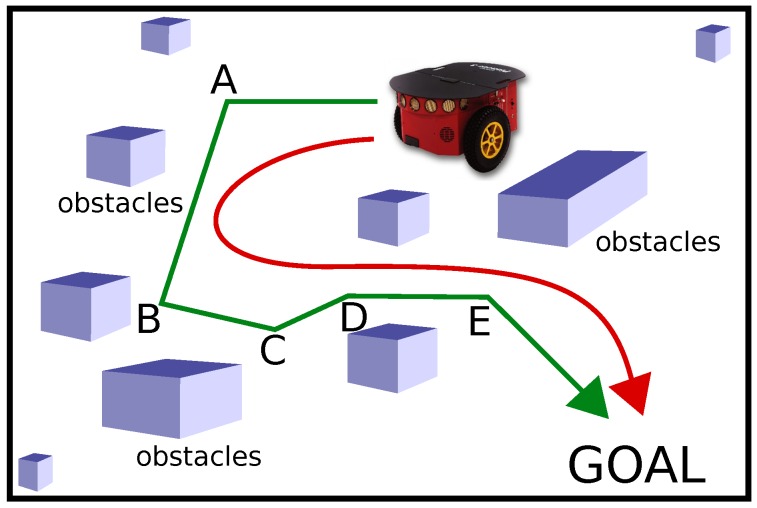
The green path is a path consisting of straight lines and sharp turns at points A, B, C, D, and E. A smooth and continuous path is shown in red color.

Most of the path planning algorithms generate a path consisting of straight lines and sharp turns. As an example, Figure 1 shows a robot and its goal location. The green path is a path consisting of straight lines and sharp turns at points A, B, C, D, and E. Such path is not desired for robot motion as the robot cannot make these sharp turns suddenly, and needs to slow down. In the case of a robotic wheelchair operated by an injured person or patient, such sudden stops and sharp turns are not favorable and may even cause injuries or induce unpleasant feelings in the person. Moreover, from an external person’s point of view in the same environment such erratic robot motion is unnatural, and it is difficult to predict the robot’s next position to avoid collision. In some cases, depending on the kinematics of the robot, it might even be difficult to execute some of these sharp turns. Such paths are also not suitable for robots delivering delicate, precious, or dangerous items.

A smooth and continuous path is desirable for robot navigation as shown in red color in Figure 1. Such path avoids abrupt and sharp turns, and the robot can maneuver without stopping. Path smoothing is an important problem in service robots and the smooth paths must satisfy certain constraints like continuity and safety. The continuity problem mainly refers to the geometric continuity in terms of tangential or curvature continuity. The safety check ensures that the smooth path is sufficiently far from the obstacles. Robot motion kinematics is another factor which must be considered while smoothing the paths.

In this paper, we review the state-of-the-art approaches used for smoothing the paths of the mobile robots. We not only summarize the various algorithms, but also provide a concise mathematical description about the particular techniques. We discuss the novelty, advantages, and limitations of each method. At last we discuss some of the main challenges in path smoothing.

## 2. A Short Note on Path Continuity

The smoothness of a path is generally expressed in terms of continuity [27]. Continuity is of two types: (a) Geometric continuity ($G^{i}$), and (b) Parametric continuity ($C^{i}$). Geometric continuity ensures that the endpoints of the various segments of the path meet, and the tangent vector’s directions are equal. Parametric continuity ensure that the endpoints of the various path segments meet, and tangent vector’s direction and magnitudes both are equal. Roughly speaking, parametric continuity ($C^{i}$) implies geometric continuity ($G^{i}$), but not vice-versa.

**Figure 2:**
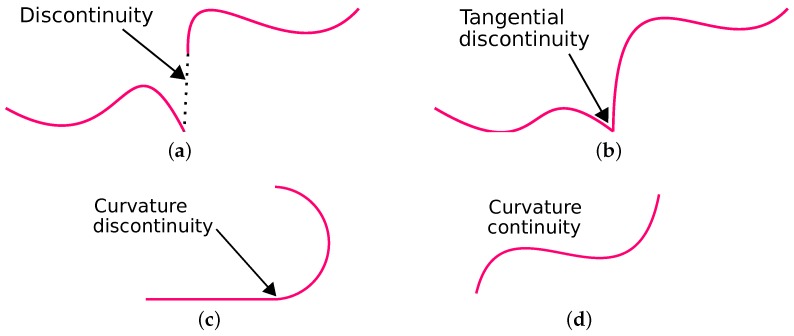
Parametric continuity. (**a**) Discontinuous curve segments. (**b**) $C^{0}$ continuity. (**c**) $C^{1}$ continuity. (**d**) $C^{2}$ continuity.

Two curves are $C^{i}$ continuous at a point *p* if the *i*th derivatives of the two curves are also equal at the point *p*. If segments of the two curves are $C^{i}$ continuous at point *p*, then they are also $C^{k}$ continuous ∀k≤i. In general, a curve *s* has $C^{n}$ continuity if it’s *n*th derivative $\frac{d^{n}s}{dt^{n}}$ is also continuous. Figure 2a shows a discontinuous path. A $C^{0}$ continuous path is shown in Figure 2b and it connects all the points between the start and the goal location such that there is no discontinuity. However, there is a slope discontinuity at the point joining the two path segments. A $C^{1}$ continuous path is shown in Figure 2c is also $C^{0}$ continuous and also preserves the tangency at the point of joint. In other words, the path matches first differential values at each point in the path. However, the curvature of the two segments are not continuous. It is evident that in Figure 2c that the straight segment has no curvature, while the curved segment has a finite curvature. Hence, the curvature suddenly bumps from zero to a finite value at the point joining the two segments shown in Figure 2d. A $C^{2}$ continuous path is shown in Figure 2d and preserves the second order differential values at each point in the path. Therefore, $C^{1}$ continuous path is smoother than $C^{0}$ continuous at the point joining the two curves. Similarly, $C^{2}$ continuous is smoother than $C^{1}$ continuous curve at the joint.

In terms of robot path planning and motion, the terms parametric continuity ($C^{i}$) and geometric continuity ($G^{i}$) are both found in the literature. As discussed in [28,29,30], parametric continuity means smoothness both of the curve and of its parameterization. Geometric continuity simply means the smoothness of the track that the robot traverses. For example, $C^{1}$ continuity means continuity of the tangent vector, while $G^{1}$ continuity means continuity of slope; $C^{2}$ continuity means continuity of the acceleration vector, while $G^{2}$ continuity means continuity of the curvature. In terms of robot motion, the $C^{1}$ continuous motion preserves velocity, whereas $C^{2}$ continuous path preserves acceleration. For robot path planning, what matters is mainly the path’s $C^{1}$ or $C^{2}$ continuity. Higher orders of continuity like $C^{3}$ continuity or higher deals with surface continuity and find usage mainly in CAD/CAM based design applications.

## 3. Interpolation Based Path Smoothing

In literature, interpolation technique was first proposed by E. Warning [31,32]. Precisely, given m+1 pairs $\left(x_{i},y_{i}\right)$, the problem consists of finding a function ψ=ψ(x) such that $\psi \left(x_{i}\right)=y_{i}$ for $i=0,\cdots ,m,y_{i}$ being some given values, and say that ψ interpolates $\left\{y_{i}\right\}$ at the nodes $\left\{x_{i}\right\}$. We speak about polynomial interpolation if ψ is an algebraic polynomial, trigonometric approximation if ψ is a trigonometric polynomial, or piecewise polynomial interpolation (or spline interpolation) if ψ is only locally a polynomial.

### 3.1. Polynomial Interpolation

In literature, the two major polynomial interpolation techniques found for path smoothing use Lagrange’s interpolating polynomial and Hermite’s interpolating polynomial which are discussed below.

The Lagrange interpolating polynomial [33] is the polynomial P(x) of degree ≤(n−1) that passes through the *n* points $\left(x_{1},y_{1}=f\left(x_{1}\right)\right),\left(x_{2},y_{2}=f\left(x_{2}\right)\right),\cdots ,\left(x_{n},y_{n}=f\left(x_{n}\right)\right)$, and is given by,

(1) $$P\left(x\right)=\sum_{j=1}^{n}P_{j}\left(x\right),$$

where,

(2) $$P_{j}\left(x\right)=y_{j}\prod_{k=1,k\neq j}^{n}\frac{x-x_{k}}{x_{j}-x_{k}}.$$

Another interpolation technique is the Hermite’s interpolating polynomial. Let p(x) be an *n*th degree polynomial with zeros at $x_{1},\cdots ,x_{n}$. Then, the fundamental Hermite interpolating polynomials [34,35] of the first and second kinds are defined by,

(3) $$h_{i}^{\left(1\right)}\left(x\right)=\left[1-\frac{p^{\prime \prime }\left(x_{i}\right)}{p^{\prime }\left(x_{i}\right)}\left(x-x_{i}\right)\right]{\left[p_{i}\left(x\right)\right]}^{2}$$

and

(4) $$h_{i}^{\left(2\right)}\left(x\right)=\left(x-x_{i}\right){\left[p_{i}\left(x\right)\right]}^{2},$$

for i=1,2,⋯,n, where the fundamental polynomials of Lagrange interpolation are defined by,

(5) $$p_{i}\left(x\right)=\frac{p(x)}{p^{\prime }\left(x_{i}\right)\left(x-x_{i}\right)}.$$

Path smoothing using interpolation is very old class of algorithms [36]. Two major limitations of interpolation techniques are: (a) high computational costs, and (b) Runge’s phenomenon [37] which is a classic illustration of polynomial interpolation non-convergence.

In a recent work ([38,39]) proposed by S.R. Chang and U.Y. Huh, a QPMI (Quadratic Polynomial and Membership Interpolation) algorithm was proposed, which avoids Runge’s phenomenon [37] and the weakness of spline interpolation, by creating a $G^{2}$ continuous path using just the quadratic polynomials and membership functions. In [39], S.R. Chang et al. propose a collision free continuous $G^{2}$ path using interpolation. However, their methods required explicit collision detection checks, and reprogramming of smooth paths which can be expensive in case of crowded environments. Other approaches involve using quintic polynomials [40]. A summary of work related to interpolation can be found in [29].

### 3.2. Bézier Curve

**Figure 3:**
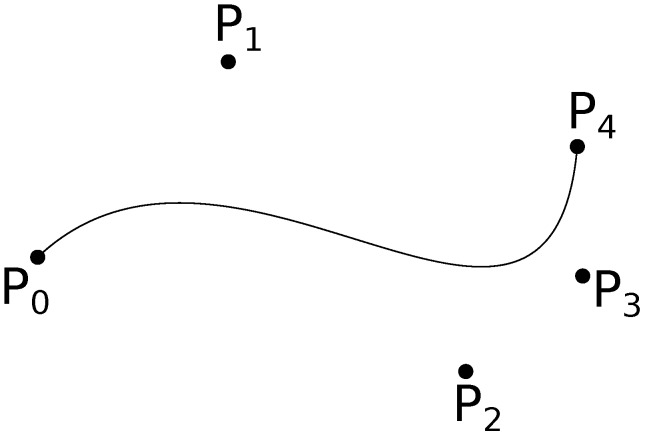
Bezier curve through control points $P_{0},P_{1},\cdots ,P_{4}$.

These parametric curves make use of ‘control points’ to define the shape as shown in Figure 3. At their core, they make use of Bernstein polynomial functions [41]. Given a set of n+1 control points $P_{0},P_{1},\cdots ,P_{n}$, the corresponding Bézier curve (or Bernstein-Bézier curve) [42,43] is given by,

(6) $$C\left(t\right)=\sum_{i=0}^{n}P_{i}B_{i,n}\left(t\right),$$

where, $B_{i,n}\left(t\right)$ is a Bernstein polynomial [41] and t∈[0,1]. A Bernstein polynomial of degree n is defined by,

(7) $$B_{i,n}\left(t\right)=\left(\begin{array}{c} n\\ i \end{array}\right)t^{i}{\left(1-t\right)}^{n-i},$$

where,

(8) $$\left(\begin{array}{c} n\\ i \end{array}\right)=\frac{n!}{i!(n-1)!}.$$

Work in [42] proposes two path planning algorithms which makes use of Bezier curves generated using proper positioning of the control points for a mobile robot to navigate in a corridor with constraints. In [44], a smooth path generation for automated vehicle using Bezier curve is proposed for both 2D and 3D case. Work in [42,45] discusses the optimized placement of control points. An interesting work in [46] uses Bezier curves as transition curves (described in Section 6) to join a straight segment with a curved segment, and also in generating clothoidal paths [47,48] demonstrating their modularity and extensibility. Trajectory generation for vehicles in urban environments has been discussed in [49,50,51]. Some researchers have used Bezier curves for automatic parking of vehicles [52].

### 3.3. Cubic Splines

A spline [53,54] is a piecewise polynomial function that can have a locally very simple form, yet at the same time be globally flexible and smooth. Splines are very useful for modeling arbitrary functions, and are used extensively in computer graphics. In interpolating problems, spline interpolation is often preferred to polynomial interpolation because it yields similar results, even when using low degree polynomials, while avoiding Runge’s phenomenon for higher degrees.

The two important characteristics of interpolatory cubic splines [35] are: (1) they are the splines of minimum degree that yield $C^{2}$ approximations; and (2) they are sufficiently smooth in the presence of small curvatures. Let us thus consider, in [a,b], n+1 ordered nodes $a=x_{0}<x_{1}<\cdots <x_{n}=b$ and the corresponding evaluations $f_{i},i=0,\cdots ,n$. The aim is to provide an efficient procedure for constructing the cubic spline interpolating those values. Since the spline is of degree 3, its second-order derivative must be continuous. By introducing the following notation $f_{i}=s_{3}\left(x_{i}\right),\;m_{i}=s_{3}^{\prime }\left(x_{i}\right),\;M_{i}=s_{3}^{\prime \prime }\left(x_{i}\right),\;i=0,\;\cdots ,\;n$, the cubic spline interpolation is given by,

(9) $$s_{3,i-1}\left(x\right)=M_{i-1}\frac{{\left(x_{i}-x\right)}^{3}}{6h_{i}}+M_{i}\frac{{\left(x-x_{i-1}\right)}^{3}}{6h_{i}}+C_{i-1}\left(x-x_{i-1}\right)+{\tilde{C}}_{i-1}$$

where, $h_{i}=x_{i}-x_{i-1},i=0,\cdots ,n$ and,

(10) $${\tilde{C}}_{i-1}=f_{i-1}-M_{i-1}\frac{h_{i}^{2}}{6},C_{i-1}=\frac{f_{i}-f_{i-1}}{h_{i}}-\frac{h_{i}}{6}\left(M_{i}-M_{i-1}\right),$$

$M_{i}$ can be calculated from the M-continuity system:

(11) $$\mu_{i}M_{i-1}+2M_{i}+\lambda_{i}M_{i+1}=d_{i},\;i=0,\cdots ,n.$$

### 3.4. B-Spline

A B-spline is a generalization of the Bézier curve. Given *m* real values $x_{i}$, $x_{0}\leq x_{1}\leq \cdots \leq x_{m-1}$, called knots, B-spline parametric curve of degree *n*,

$$S:\left[x_{0},x_{m-1}\right]\to R^{2}$$

is composed of a linear combination of basis B-splines $b_{i,n}$ of degree *n*,

(12) $$S\left(x\right):\sum_{i=0}^{m-n-2}P_{i}B_{i,n}\left(x\right),x\in \left[x_{n},x_{m-n-1}\right],$$

where, $P_{i}$ are called control points, and the m−n−1 control points form a convex hull. The m−n−1 basis B-spline of degree *n* is defined [35] as below with j=0,⋯,m−2.

(13) $$B_{j,n}\left(x\right)=\frac{x-x_{j}}{x_{j+n}-x_{i}}B_{j,n-1}\left(x\right)+\frac{x_{j+n+1}-x}{x_{j+n+1}-x_{j+1}}B_{j+1,n-1}\left(x\right).$$

**Figure 4:**
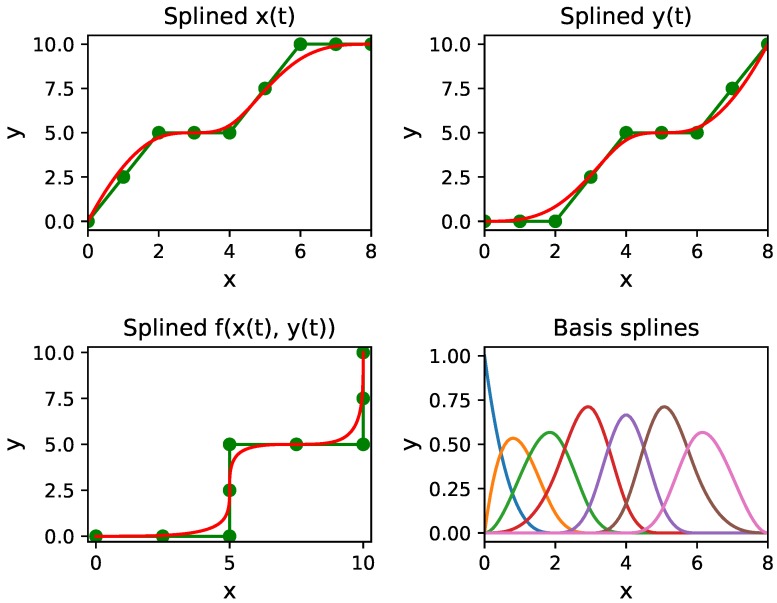
An example of path smoothing using B-Spline. The green path is the global path while the red path is the smooth path. The green dots are the control points.

Note that, when the number of control points is one more than the degree and x∈[0,1], i.e., $x_{0}=\cdots =x_{n}=0$ and $x_{n+1}=\cdots =x_{2n}=1$, the B-spline degenerates into Bezier curve [35]. Figure 4 shows B-Spline curve generated in red color along with the basis splines.

Komoriya et al. discusses trajectory design and control of a mobile robot using B-spline curves [55]. B-splines were chosen for path planning algorithm in ‘KAT-5’ vehicle [56,57] to complete the 2005 DARPA challenge [58] primarily because of the ease in which the shape of their resulting curves can be controlled. Spline interpolation has been used in the autonomous driving car ‘Stanley’ which won the DARPA challenge [59] in 2006, and ‘Odin’ [60] which secured third place in the same challenge in 2007. Berglund et al. [61] has used B-Splines for planning smooth and obstacle free paths for autonomous mining vehicles (more than 100 tonnes) operating at speeds of around 20 km/h. For vision based autonomous vehicles, a Quintic $G^{2}$-spline-based steering algorithm is proposed in [62]. B-Splines have also been used [63] for curvature continuous trajectory generation with energy minimization as the goal. In order to recover from the problems of complicated curvature functions which require many parameters improved methods using spline-based smoothing has been proposed in [64] allowing better visual representation and better data compression compared to traditional methods. In [65], a motion planner tailored for particular requirements for robotic car navigation is proposed which leverages B-spline curve properties to include vehicle’s constraint requirements, thus lowering the search dimensionality.

**Figure 5:**
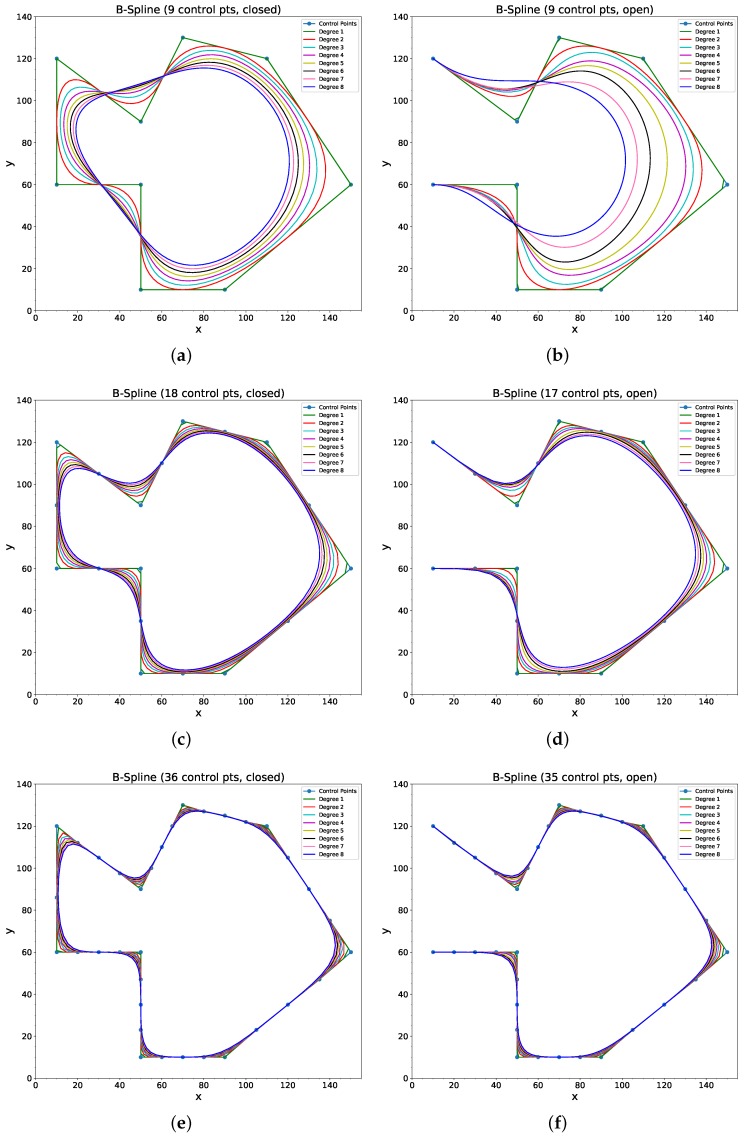
B-Spline-based robot path smoothing with varying number of control points and degrees. (**a**,**c**,**e**) shows closed path (robot returns to the same location) while (**b**,**d**,**f**) shows open paths. (**a**) Closed path, 9 control points. (**b**) Open path, 9 control points. (**c**) Closed path, 18 control points. (**d**) Open path, 17 control points. (**e**) Closed path, 36 control points. (**f**) Open path, 35 control points.

B-Splines are powerful tools for path smoothing as desired trajectory can be generated for different degrees and obstacle configuration. For example, Figure 5 shows various trajectories based on different number of control points and curve degrees. Without smoothing, the robot will encounter sharp turns at the corners. Figure 5a shows B-Spline-based trajectories with nine control points and curve degrees from 1 to 8, in a closed form (i.e., the start and end points are the same). In this configuration, the generated curves are too far from the actual points. However, by doubling the number of control points by introducing extra control points between two control points, the turns are smoothed out and the smoothed trajectories are shown in Figure 5c for various degrees. Similarly, Figure 5e shows the smooth trajectory with 36 control points. In this case, it can be seen that the smoothed trajectory keeps the straight segments straight and only smooths the sharp turns for varying degrees of the curve. Figure 5b,d,f shows the open loop versions of their respective figures on left column of Figure 5.

### 3.5. NURBS Curve

A non-uniform rational B-spline (NURBS) curve [66,67,68] is defined by,

(14) $$C\left(t\right)=\frac{\sum_{i=0}^{n}N_{i,p}\left(t\right)w_{i}P_{i}}{\sum_{i=0}^{n}N_{i,p}\left(t\right)w_{i}}$$

where *p* is the order, $N_{i,p}$ are the B-spline basis functions, $P_{i}$ are control points, and the weight $w_{i}$ of $P_{i}$ is the last ordinate of the homogeneous point $P_{i}^{w}$.

Thus, by manipulating both the control points and the weights, NURBS prove to be a very flexible tool to generate desired trajectories. Moreover, NURBS are invariant under shear, translation, rotation, scaling, as well as parallel and perspective projection [69]. NURBS based path smoothing has been applied in [70] for trajectory generation of an industrial five axis needle winding robot with optimal winding pattern in the stator slots. Apart from trajectory planning, some researchers have used NURBS curves for 3D map smoothing [71]. Work in [72] proposes an arm trajectory planner with obstacle avoidance. Similarly, Quintic NURBS has also been used for the optimal trajectory planning of manipulators in [73]. In [74,75,76], NURBS based trajectory generation for an autonomous mobile robot navigating in 3D environment has been proposed with obstacle avoidance. NURBS has also been used in swarm robotics [77], path planning of humanoid robots [78], and even segmenting unknown objects in RGB-D images for robotics tasks such as object search, grasping and manipulation [79].

The main drawbacks of using NURBS is that they require extra storage, and improper initialization of weights can lead to bad parametrization [69]. However, NURBS are still powerful with clear geometric interpretations, fast evaluation, and stable computation [80].

## 4. Path Smoothing Using Special Curves

The other segment of robot path smoothing algorithms utilize one or more curves from the family of curves which includes: parabola, ellipse, hyperbola, cardioid, limacon, hypocycloid, cycloid, pedal curves, and spirals. A detailed mathematical description of these curves is given in [81]. Here, we discuss some of the most prominent curves found in the state-of-the-art.

### 4.1. Dubin’s Curve

**Figure 6:**
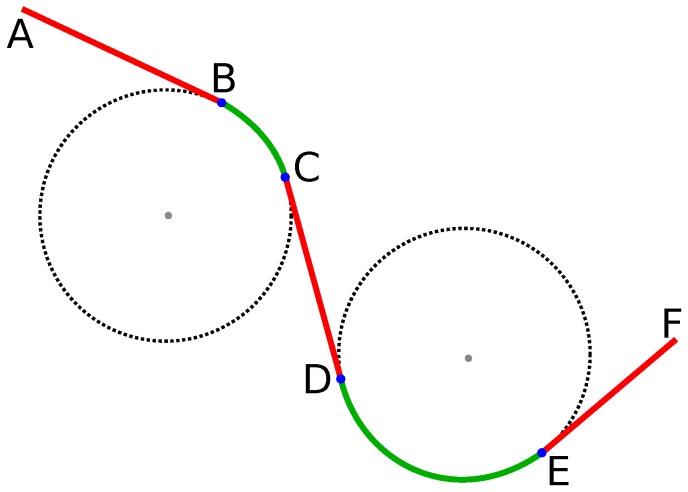
Path smoothing using Dubin’s curve. Straight segments $\bar{\mathrm{AB}}$, $\bar{\mathrm{CD}}$, and $\bar{\mathrm{EF}}$ in red color are combined with circular arcs BC and DE shown in green color.

Given two points and in a plane and a specified direction of motion, Dubins [82] in 1957 used circular arcs and straight line segments to find the shortest smooth path of bounded curvature that joins the points [13,83]. Figure 6 shows an example of path smoothing using Dubin’s curves. Segments shown in red color i.e., $\bar{\mathrm{AB}}$, $\bar{\mathrm{CD}}$, and $\bar{\mathrm{EF}}$ are the straight segments which are combined with circular arcs BC and DE shown in green color.

Since then, a number of modifications and improvements have been proposed. In [84], authors have considered using Dubin’s curve with obstacle avoidance. Unmanned Aerial Vehicle (UAVs) navigation using predictive vector field control is proposed in [85] which uses Dubin curves. Similarly, work in [86] proposes a path planning algorithm based on 3D Dubins curves for UAVs to avoid both static and moving obstacles using a variation of Rapidly-exploring Random Tree (RRT) [21] as the planner. In [87], authors have proposed an efficient two-phase approach to motion planning for small fixed-wing UAVs navigating in complex 3D environments. Path smoothing for UAV with similar goals is proposed in [88]. First, a kinematically feasible obstacle-free coarse global path is computed in a discretized 3D environment. Then, the optimal trajectory based on a set of admissible paths based on the task tree approach are generated using Dubin’s curve. Work in [89] proposes an algorithm for planning $C^{\infty }$ paths with bound curvature and curvature derivative linking two fixed (initial and final) configurations and passing through a given number of intermediate via-points. In [90] authors have used Dubins curves extended to 3D case that model the properties of turn and straight flight for wind-aware emergency landing. In agricultural robotics, Dubin’s curves have been used for coverage path planning of autonomous robotic lawn mower equipped with GPS in [91].

Dubin’s curves provide a simple yet powerful technique for real-time path smoothing as they are generally not computationally expensive. They can be combined with clothoids (discussed in Section 4.2) to satisfy different constraints. For example, Chen et.al. proposed to use Dubin’s curve in conjunction with Fermat’s spiral [92] to design a curvature continuous path in [93]. Like clothoids, Fermat’s spiral curvature changes continuously with length.

### 4.2. Clothoid

Clothoid [94], also known as Euler’s spiral or Cornu’s spiral is the curve parameterized in the complex plane of points by,

(15) B(t)=S(t)+iC(t),

where,

(16) $$\begin{array}{c} x\left(t\right)=C\left(t\right)=\int_{0}^{t}\cos\left(\frac{\pi }{2}s^{2}\right)\;ds\\ y\left(t\right)=S\left(t\right)=\int_{0}^{t}\sin\left(\frac{\pi }{2}s^{2}\right)\;ds \end{array}$$

where *C* and *S* are the Fresnel functions [95], sometimes called the Fresnel cosine integral and Fresnel sine integral.

Both Fresnel functions approach $\frac{1}{2}$ as t→∞ and so the curve slowly spirals toward $\left(\frac{1}{2},\frac{1}{2}\right)$ in the first quadrant. And by symmetry, because both functions are odd, the curve spirals toward $\left(-\frac{1}{2},-\frac{1}{2}\right)$ in the third quadrant. Cornu’s curve has the property that its curvature is proportional to the distance along the path of the curve. Hence, a vehicle traveling at constant speed will experience a constant rate of angular acceleration as it travels around the curve—this means that the driver can turn the steering wheel at a constant rate which makes the junction safer.

These properties make clothoids an attractive option for path smoothing of trajectories. In [96], path smoothing for car-like vehicle navigation has been proposed in which a continuous curvature path is generated by multiple clothoids composition and parametric adjustment. Clothoid based path smoothing has been presented in [97,98,99,100,101]. Recently, clothoids have been used in trajectory generation of autonomous Audi TTS car [102], and vehicles in VisLab Intercontinental Autonomous Challenge [103]. Some researchers have used clothoids for autonomous parallel parking with geometric continuous-curvature path planning in [104,105,106].

Brezak et al. proposed [107] a method computation of clothoid coordinates that guarantees bounded approximation error over a wide range of clothoid parameters in real-time. Clothoids have also been used in trajectory planning of vehicles at relatively large speeds than mobile robots. For instance, work in [108] proposes using dynamically feasible clothoid trajectories which provide a reliable method for representing the vehicle’s path for the next few seconds of driving. In [109] authors have considered a case of trajectory generation of vehicle consisting of a robotic walking assistant pushed by a user. In [110] motion planning algorithm is proposed for a mobile robot that reduces not only the path length, but also the curvature change along the path using clothoids. In [111] authors have considered the bounded-curvature path of the of the three-wheeled omni-directional mobile robot based on a smooth road which is described as a clothoid. Connecting the straight segments by symmetric clothoid curves at the junction has been proposed in [112]. An overtaking maneuver of mobile robot using clothoids is undertaken in [113].

Work in [114] has proposed to integrate clothoid based trajectory into the Robot Operating System (ROS) [115] framework. It should be noted that ROS is a very popular framework for developing robot applications. Integration of such path smoothing algorithms in the ROS framework should facilitate mobile robot navigation based applications.

### 4.3. Hypocycloid

**Figure 7:**
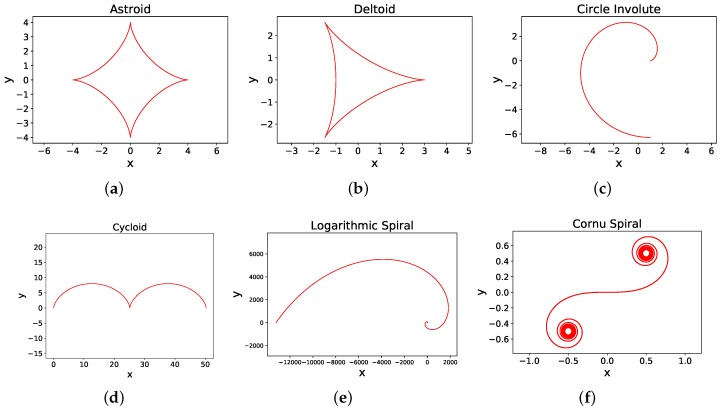
Curves for path smoothing. (**a**) Astroid. (**b**) Deltoid. (**c**) Circle Involute. (**d**) Cycloid. (**e**) Logarithmic Spiral. (**f**) Clothoid or Cornu Spiral (Euler’s Spiral).

A hypocycloid is a geometrical curve which is produced by a fixed point *P* which lies on the circumference of a small circle of radius $r_{s}$ rolling inside a larger circle of radius $R_{L}>r_{s}$ [81,116]. The rolling circle has a radius of $r_{s}$ and the large circle has a radius of $R_{L}$, and the curve is defined in general by,

(17) $$\begin{array}{c} x\left(\theta \right)=\left(R_{L}-r_{s}\right)\cos\left(\theta \right)+r_{s}\cdot \cos\left(\frac{R_{L}-r_{s}}{r_{s}}\right)\theta \\ y\left(\theta \right)=\left(R_{L}-r_{s}\right)\sin\left(\theta \right)-r_{s}\cdot \sin\left(\frac{R_{L}-r_{s}}{r_{s}}\right)\theta \end{array}$$

where, η is the number of cusps and ξ is the ratio of the radius of the rolling circle to the radius of the large circle, i.e., $\xi =\frac{r_{s}}{R_{L}}$. A cusp is defined as the sharp corner where the curve is not differentiable. Figure 7b shows a 3-cusped hypocycloid called as a deltoid or tricupsoid. A 4-cusped hypocycloid called as an astroid is shown in Figure 7a.

In general, to obtain *n* cusps in a hypocycloid, the radius of smaller circle is set to $r_{s}=\frac{R_{L}}{n}$, as *n* rotations of the smaller circle brings it back to the original position, generating *n* cusps while traversal [81]. For a deltoid, $R_{L}=3\times r_{s},\;\xi =\frac{1}{3}$ and for astroid, $R_{L}=4\times r_{s}$, and $\xi =\frac{1}{4}$.

**Figure 8:**
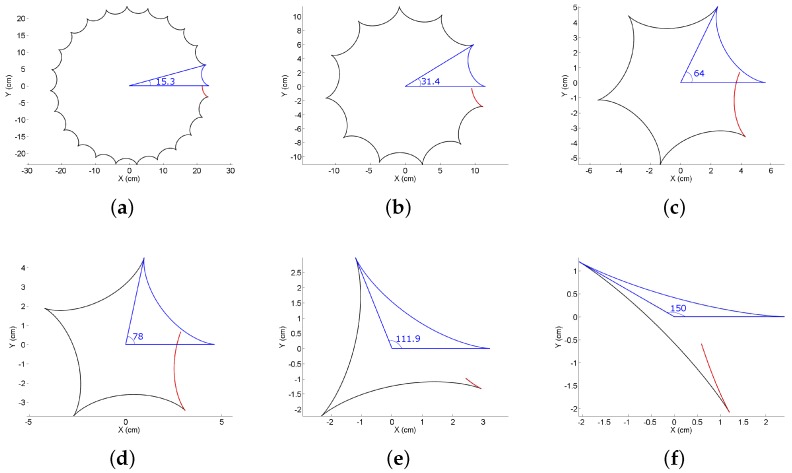
SHP [117] generation with different angles. The first segment of the curve in blue is taken to generate SHP, whereas other curve segments in black and red are ignored. (**a**) θ=15.3${}^{\circ }$. (**b**) θ=31.4${}^{\circ }$. (**c**) θ=64${}^{\circ }$. (**d**) θ=78${}^{\circ }$. (**e**) θ=111.9${}^{\circ }$. (**f**) θ=150${}^{\circ }$.

Recently, a new approach of Smooth Hypocycloidal Paths (SHP) has been proposed in [117]. An important characteristic of hypocycloids is that they can be generated for any angle as shown in Figure 8. Figure 8 shows hypocycloid curve generation for various angles. This property is used in [117] for path smoothing with an aim to keep the straight segments straight and only smooth the points of sharp turns. In [118], an extension of SHP has been proposed in which SHP has been proposed as an ‘extension’ or a ‘plug-in’. Any of the traditional path planning algorithms can be used for overall planning and the sharp turns are detected and smoothed using hypocycloidal curves. Similarly, other curves like circle involute (Figure 7c), cycloid (Figure 7d), logarithmic spiral (Figure 7e), and clothoids (Figure 7f) can also be used for smoothing and a detailed mathematical description can be found in [81].

## 5. Optimization-Based Path Smoothing

Path smoothing has also been considered as a function optimization problem guiding a mobile robot on the lane and avoiding obstacles through minimizing several parameter constraints like speed, rollover constraints, acceleration, jerk, and others [119,120,121,122].

Work in [123] proposes a convex elastic smoothing heuristic algorithm for trajectory smoothing and speed optimization for mobile robots with car-like dynamics. The key feature of the algorithm is that the optimization problem can be solved via convex programming, making it fast. In [124], authors have proposed an algorithm for a non-holonomic wheeled vehicle operating in a semi-structured environment. First, the algorithm computes offline a finite set of feasible motions connecting discrete robot states to construct a search graph. Later, the motion primitives based on Bezier curves are generated by solving the constrained optimization problem.

In this regard “Time Elastic Band” planner (TEB Planner) it is worth mentioning [125,126,127,128,129] as it is available with ROS integration [130,131]. Timed Elastic Band locally optimizes the robot’s trajectory with respect to trajectory execution time, separation from obstacles and compliance with kinodynamic constraints at runtime. The TEB planner optimizes robot trajectories by subsequent modification of an initial trajectory generated by a global planner. The objectives considered in the trajectory optimization include the overall path length, trajectory execution time, separation from obstacles, passing through intermediate way points, compliance with the robots dynamic, kinematic and geometric constraints, and dynamic obstacles. It also allows efficient online motion planning of car-like robots.

Some of these optimization based approaches have been used practically in the DARPA Grand Challenge. Work in [132,133] proposes an optimization based navigation algorithm for the DARPA Grand Challenge. The authors address the path planning problem with a nonlinear optimization method running in real-time. An optimization problem is continually solved to find a time-optimal, dynamically feasible trajectory from the vehicle’s position to some receding horizon ahead (20 m–70 m forward).

Similarly, in [134] authors describe a practical path-planning algorithm for an autonomous vehicle operating in an unknown semi-structured (or unstructured) environment, where obstacles are detected online by the robot’s sensors. The first phase uses a variant of A* search to obtain a kinematically feasible trajectory. The second phase then improves the quality of the solution via numeric non-linear optimization, leading to a local (and frequently global) optimum. Work in [135,136] presents an algorithm for trajectory planning used on-board the vehicle that completed the 103 km of the Bertha-Benz-Memorial-Route fully autonomously. In the algorithm, the constraints are carefully designed to ensure that the solution converges to a single, global optimum. In [137] authors propose a motion planner for autonomous vehicles based on the idea of a on-road state lattice. A focused search is performed in the previously identified region in which the optimal trajectory is most likely to exist.

A comparison of various trajectory smoothing methods in terms of advantages and disadvantages is given in Table 1.

## 6. A Note on Transition Curves

There are many algorithms in the literature (for example Dubin’s curves [82], SHP [117], etc.) which ensure a $C^{1}$ continuity in which the tangential constraint is satisfied, however, higher orders of continuity like $C^{2}$ curvature continuity is not guaranteed. As explained in [117], although $C^{1}$ continuity is enough for mobile robots navigating at low speed, in case of a robot traveling at high speed with acceleration, it is not enough. In these cases, a transition between the straight line and the curved section is required. This is a well studied problem and various solutions are available. In fact, smoothing out a straight section of track to a curved section while maintaining curvature continuity using transition curves [138] has extensively been studied for building railway tracks and highway roads. We briefly discuss transition curves, as their solutions in parametric forms can easily be obtained and computed.

Transition curves [138] connect a straight segment of the path at one end to the curve at the other end. Hence, radius of curvature changes from zero on the straight segment to a finite value of the curve, at a uniform rate. It eliminates the kink generated by directly connecting the straight and the curve section [117]. Transition curves have the following important properties desirable for robot motion: (a) They are tangential to the straight line of the path, i.e., curvature at start is zero. (b) It joins the circular curve tangentially. (c) It’s curvature increases at the same rate. Transition curves have rigorously been studied for $C^{2}$ continuity in many works [139,140,141], and can also provide smooth $C^{2}$ transition between two straight lines [117].

**Figure 9:**
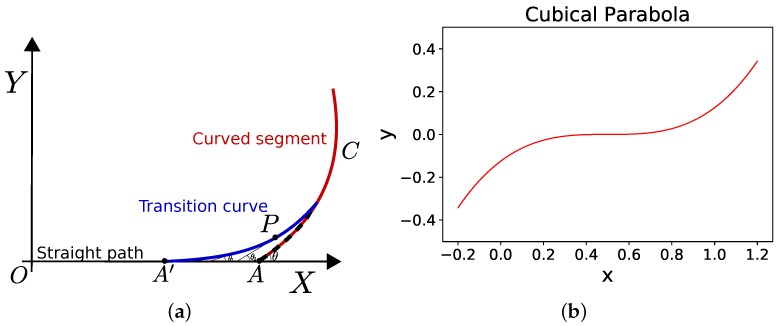

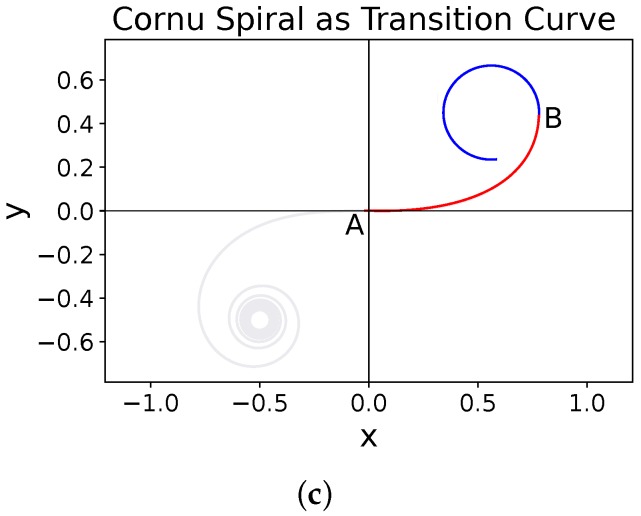
Transition Curve. (**a**) Point A is shifted back to A’ which is the starting point of the transition curve shown in blue. There is a gradual increase in curvature so that there is no sudden kink or jerk. (**b**) Cubical parabola as a transition curve. (**c**) Cornu Spiral or clothoid as a transition curve.

Figure 9a shows a curved path in red meeting the straight segment at point A. To generate transition curve, point A is shifted back to A’ which is the starting point of the transition curve shown in blue. Although different types of curves can be used to generate transition curves, clothoids (i.e., Euler’s curve or cornu spiral shown in Figure 9c) are the most common [138]. Cubical parabola shown in Figure 9b can also be used. Taking into account linear increase of curvature from zero on the straight segment to the curvature of the hypocycloid, rate of change of angle at any point (P) on the transition curve of length *L* (shown in blue in Figure 9a) is given by,

(18) $$\begin{array}{c} \frac{d\theta }{ds}=\frac{s}{R_{L}\cdot L}, \end{array}$$

where *s* is the length of the transition curve from point A’ to P in Figure 9a. Integrating Equation (18), we obtain,

(19) $$\begin{array}{c} \theta =\frac{s^{2}}{2R_{L}\cdot L}. \end{array}$$

During the initial condition (on straight segment) when s=0, θ is also 0. As explained in [117], apart from polar coordinates, the equation of transition curve in parametric form is given as [138,142],

(20) $$\begin{array}{l} x=s-\frac{s^{5}}{40R_{L}^{2}L^{2}},\\ y=\frac{s^{3}}{6R_{L}}-\frac{s^{7}}{336R_{L}^{3}L^{3}}. \end{array}$$

Similarly, Figure 9c shows using a clothoid in red color for smooth transition from point A to B to meet the curve in blue color. Authors in [117] have described using transition curves for mobile robot navigation applications.

## 7. Robot Trajectories with Obstacle Avoidance

**Figure 10:**
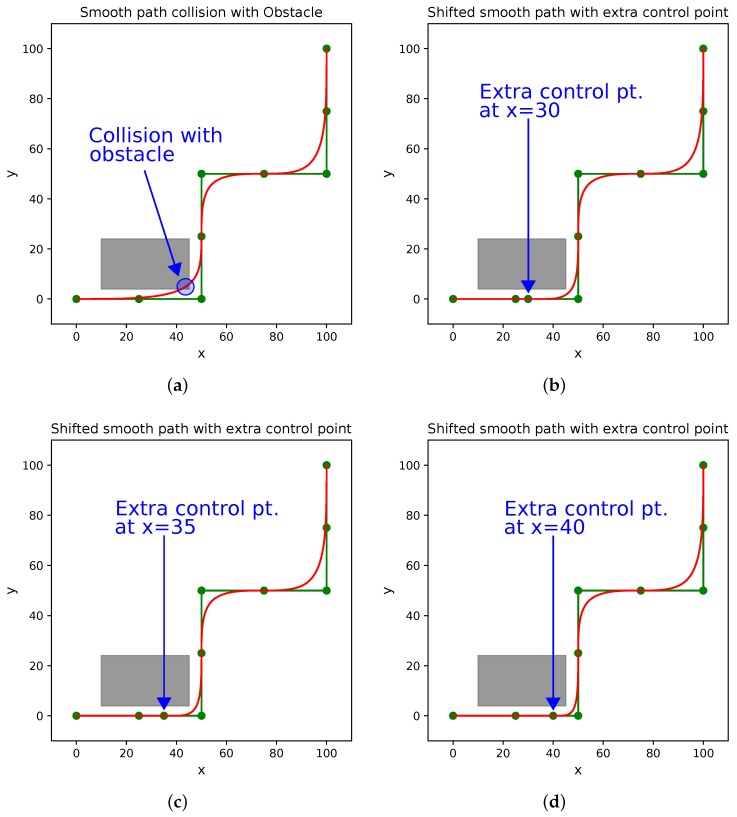
Problem of smooth trajectory generation while avoiding collision with obstacles with the case of B-Spline-based smoothing. (**a**) Smooth path in red color collides with the obstacle. (**b**) Inclusion of extra control point at x=30 avoids collision. (**c**) Smooth path with extra control point at x=35. (**d**) Smooth path with extra control point at x=40.

There is a huge plethora of research work in static and dynamic obstacle avoidance of mobile robots. This section limits the review to obstacle avoidance in the context of smooth trajectory generation. Many works in the state-of-the-art considers a static scenario in which the positions of the obstacles are well known and smooth paths are generated. However, in real environments there are moving entities like people and other robots in vicinity and smoothing must be done in real-time to avoid collision while still traversing a smooth path. Even in the case of outdoor robots and autonomous vehicles, there is a constant challenge from multiple entities like pedestrians, cyclists, and other vehicles. Thus, there is a gap between global path planning and real-time sensor-based robot control. This is graphically explained by taking an example in Figure 10 in which the gray rectangle represents an obstacle while the green path is the non-optimized global path. B-spline-based smooth trajectory is generated and shown in red color. In Figure 10a, the smoothed path collides with the obstacle and not suitable. Hence by introducing additional control points at x=30, x=35, and x=40, the smooth path can be adjusted to avoid collision as shown in Figure 10b,c,d, respectively. This is easy in case of static obstacles. However, dynamic obstacles have to be tracked for speed and orientation, and the control points needs to be fixed at correct places to generate the trajectory (for example in case of using B-Splines).

In this regard, one of the significant early works was proposed by S. Quinlan and O. Khatib in “Elastic Bands” planner [143,144]. An elastic band is a deformable collision-free path whose initial shape is the planner’s smooth global path. The elastic band continues to deform as changes in the environment are detected by sensors, enabling the robot to accommodate uncertainties and react to unexpected and moving obstacles. This is done in real time and the smooth paths maintains a safe threshold distance from the obstacles [143]. Later this approach was extended to non-holonomic kinematics [145,146,147], robotic systems with many degrees of freedom [148], and dynamics obstacles [149]. However, these works does not take any dynamic constraints of the underlying robot into account directly. An improved method build upon [143] called timed elastic band [126,127] considers temporal aspects of the motion in terms of dynamic constraints such as limited robot velocities and accelerations, and the problem is formulated in a weighted multi-objective optimization framework, and is suitable for high dimensional state spaces. The disadvantage of timed elastic band local planner is that it often gets stuck in a locally optimal trajectory as they are unable to transit across obstacles. This limitation was addressed in [125,128], in which, a subset of admissible trajectories of distinctive topologies are optimized in parallel. The local planner is able to switch to the current globally optimal trajectory from the candidate set. The extension to robots with car-like motion model was proposed in [129,130].

The controllability and ease of smooth trajectory generation while avoiding the obstacles is also important. Splines reviewed in Section 3.4 are potential tools in this respect. The *culebra* (Spanish for “snake”) algorithm proposed in [57] for the KAT-5 vehicle [56]. In it, a centerline is first generated from the waypoints. Additional equidistant control points are inserted between the original waypoints and a cubic B-spline is drawn fitting the control points until no obstacles were found along the resulting path, iteratively. In the vehicle Stanley [59], if an obstacle is encountered, the algorithm plans a smooth change in lateral offset that avoids the obstacle and the trajectory can be safely executed. Planning in lateral offset space also has the advantage that it gracefully handles GPS error.

A log-space solution for robotic path planning with harmonic functions is proposed in [150]. The log-space solution rapidly produces smooth obstacle-avoiding trajectories, and supports planning in exponentially larger real-world robotic applications. Similarly, work in [151] proposes a real-time path planner for a smart wheelchair using harmonic potentials. Voronoi and potential maps have been used for safe trajectory generation in [152]. In [153], collision avoidance considering the shape, kinematics, and dynamics of a mobile robot is presented. A Virtual Force Field (VFF) method was proposed in [154] for real-time obstacle avoidance approach for mobile robots. The virtual force field method integrates certainty grids for obstacle representation and potential fields for navigation, suitable for inaccurate sensor data and sensor fusion. While the VFF method provides superior real-time obstacle avoidance for fast mobile robots, some limitations concerning fast travel among densely cluttered obstacles were addressed in Vector Field Histogram [155] method which was further developed in VFH+ algorithm [156] which provides smoother robot trajectories and greater reliability, VFH∗ algorithm [157] (and variants [158]) which also provides a verification that the selected trajectory avoids obstacles, and VFH*TDT (VFH* with Time Dependent Tree ) algorithm [159].

In [160], a curvature velocity method based on a probabilistic 3D occupancy and velocity grid is proposed for dynamic scenarios. Apart from curvature velocity and potential methods, dynamic window approach based obstacle avoidance [26,161,162], and nearness diagram [163,164,165,166,167] based algorithms have also shown promising results for obstacle avoidance. It is worth mentioning that apart from smooth and safe trajectory generation avoiding obstacles, other factors like path length, accuracy, control, computational cost, and reliability in uncertain scenarios are also important. A review of obstacle avoidance algorithms for mobile robots can be found in [168].

## 8. Challenges

**Figure 11:**
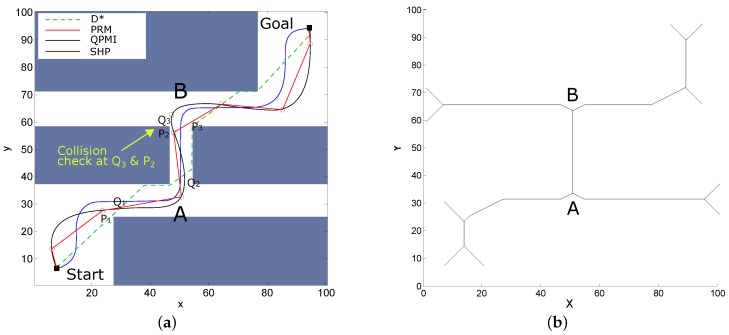
Safety and user experience while smoothing paths. (**a**) Paths generated by D* [19], PRM [25], QPMI [39], and SHP [117] in dotted green, red, black, and blue colors, respectively. (**b**) The skeleton path of the environment of Figure 11a.

The research in trajectory smoothing has been successful at various fronts, nevertheless, several challenges still remain and are summarized below:

1. **Trajectory smoothing in dynamic environments**: One of the biggest constraint in these terms is the speed of detection of dynamic entities which is directly affected by the total number of entities tracked. Hence, in an environment like an open public space, there are a lot of dynamic entities for the mobile robot to track which consumes a lot of time. This has an adverse effect on the trajectory smoothing process as the robot ends up stopping suddenly or considerably reducing the speed while trying to select the best possible alternate trajectory among the potential candidates. It is important to accurately track these dynamic entities in conjunction to path smoothing. This is more challenging in autonomous self driving cars at high speeds. Work in [169] has succinctly summarized the challenges in pedestrian detection considering the resolution, range, and field-of-view of various on-borad sensors like radar, lidar, or omni-directional cameras with various types of hardwares. In addition, work in [170] has focused the survey of challenges of pedestrian detection with vision based sensors which are very prominent recently. In adverse conditions like low illumination, night, snowfall, and rain, it is further difficult to detect the dynamic entities while work has been done in this regards by using thermal images [171]. In both indoor and outdoor environments, occlusion is another big hindrance with dynamic entity detection and solutions have been proposed [172,173], although it still is an open problem.
2. **Fusing trajectory smoothing into SLAM process**: SLAM in an indispensable module for any mobile robot. Even autonomous vehicles need to localize themselves in the environment and build or update their map using perceptive sensors like GPS, cameras, or range sensors. SLAM models the uncertainty of the robot motion and the sensor errors to come up with the most optimum state estimation [5,174]. Trajectory generation is directly linked with currently estimated state and perception and hence fusing it in the SLAM module is beneficial. Currently, it seems like SLAM module is decoupled from the trajectory smoothing module. However, the two modules must work side-by-side to update the map with the new obstacles or entities and generate real-time smooth trajectories on the fly. Work in [175] presents a motion planning algorithm considering both the uncertainty caused by robot and dynamic entities. The motion of dynamic entities are predicted using a local planner, and the uncertainty along the predicted trajectory is computed based on Gaussian propagation. In this regard, a relative continuous-time SLAM has been proposed by Anderson et al. [176] which uses weights on cubic B-splines to represent continuous state variables. Only the local weights are adjusted during optimization, while implicit trajectory prior is arbitrary. In [177], a Simultaneous Trajectory Estimation and Mapping (STEAM) is proposed which uses Gaussian Process (GP) regression instead of cubic B-splines. It interpolates between conventional state parameterizations at certain key times. When applied in SE3, this parameterization can represent realistic probabilistic trajectories obeying nonlinear, nonholonomic motion models. Although slow for dense kernels, a careful selection can result in realistic sparse GP kernels that are very fast. A non-uniform sampling of the trajectory representation over the sliding window with continuous correction is presented in [178]. In [179], a dense map-centric SLAM method based on a continuous-time trajectory is proposed which removes the need for global trajectory optimization by introducing map deformation. Some other recent significant works in continuous-time SLAM are [180,181,182,183], while a broad overview of challenges in SLAM can be found in [184].
3. **Operator in the loop, Safety, and User Experience**: In case of tele-operated robots, currently, the trajectory generation and control lacks the input from the operator. This is more important in case of autonomous vehicles to feedback the planned trajectory to the driver, and generate smooth trajectories based on driver’s intentions. This requires active feedback mechanisms and integration of human-robot integration [185,186,187]. Researchers have proposed work in this regard in [188,189] by proposing a trajectory planning algorithm that ‘adapts’ to traffic on a lane-structured infrastructure such as highways. In many researches, the emphasis has been on the mathematical completeness of the system while the user-experience seems to get ignored. For instance, in case of an autonomous robot wheelchair used in hospitals, safety is important and the wheelchair must not come close to either of the walls. Hence, in this particular scenario, it is important that the robot wheelchair moves in nearly a straight line in the center lane of the passage. However, aiming for $C^{2}$ or higher continuities, many algorithms generate a path which brings the robot close to either of the walls. For example, Figure 11a shows the results of paths generated by D* [19], PRM [25], QPMI [39], and SHP [117] in dotted green, red, black, and blue colors, respectively (results reproduced from [117]). The curve in black is $C^{2}$ continuous, however, it brings the robot too close to the obstacles at points $Q_{2}$ and $Q_{3}$. On the other hand, SHP [117] curve in blue keeps the robot sufficiently far from the obstacles and keeps straight segments of the path straight considering the input from operators of robot wheelchair. Such safe paths are easy to generate particularly on grid-maps by using Voronoi paths [190], or using thinning algorithms [191,192] or skeleton maps [193,194]. Figure 11b shows the skeleton path of the environment shown in Figure 11a. In conjunction to the previous point, the feedback from the operator must be fused, and operator intentions must be anticipated to generate smooth paths. With the advent of autonomous cars and platforms like fully autonomous robotic wheelchair, the concept of user experience especially in terms of *passenger comfort* is being re-evaluated. This is a relatively new area of research with a strong correlation with smooth trajectory generation, and some promising research has been proposed in [195,196,197,198,199,200].

## 9. Discussion and Conclusions

In this paper, we summarized the various path smoothing techniques and algorithms present in the state-of-the-art. With the advent of autonomous robots, and self driving cars, optimal trajectory generation and smoothing becomes an important field of research. We presented the actual algorithm with concise mathematical description and then discussed the work presented by various researchers along with their novel points. The paper discussed the advantages and disadvantages of the various approaches. Since safe traversal of smooth trajectory is important, we reviewed important obstacle avoidance methodologies in mobile robotics. Finally, since there is an ongoing research in this important field, the paper discussed some of the important challenges in the field in terms of trajectory smoothing in dynamic environments, fusing trajectory smoothing in SLAM process, the importance of operator in the control loop, and safety and use experience. The review covered topics in continuous-time SLAM and simultaneous trajectory estimation and mapping with collision detection. This review did not cover the actual integration of path smoothing algorithms with the planning algorithms like A* or PRM planner. Actually executing the smooth trajectory by a robot depends on many factors like the motion model (ex. differential drive or car-like model) of the robot, its kinematic constraints and others. Moreover, the actual execution of obstacle detection and avoidance while executing the smooth trajectories also depends on the sensors attached to the robot. The smooth paths generated by various methods in the state-of-the-art are generally evaluated in terms of the continuity parameters which were briefly covered in this review.

It is anticipated that we will see an influx of more and more autonomous mobile robots and vehicles working in human-centric environments. In such scenarios, research in optimal trajectory generation and smoothing is expected to see solutions for the current problems and advance further. In that context, the present work is expected to provide readers with a thorough overview of the state-of-the-art and challenges to actively pursue research and propose novel solutions.

## Figures and Tables

**Table 1:** A comparison of various trajectory smoothing techniques.

Classification	Main Advantages (+) and Disadvantages (−)
Dubins Curve	+ Fast to compute for given configuration of obstacles.
	+ Dubin’s curves are easy to compute even on low spec hardware.
	+ Shortest paths are assured.
	− These curves do not have curvature continuity.
	− Robot will experience a jerk at the point of transition of straight line and circle.
Bezier Curve	+ Bezier curves have low computational cost.
	+ Control points can generate curve of desired characteristic.
	+ Bezier curves can be connected with each other to get desired shape.
	− With increasing degree of curves, computation costs increase.
	− Difficult to adjust for curves with higher degrees.
	− Global waypoints affect the entire curve
	− It might be difficult to place control points.
Splines	+ Splines have low computational cost.
	+ They can easily provide $C^{2}$ continuity desired for robots.
	+ Knots can easily control the shape of splines.
	− It might be difficult to balance the trade-off between continuity and desired shape.
NURBS	+ NURBS are easy to compute, with fast and stable computation.
	+ They can be very flexible to generate desired trajectories.
	+ They are invariant under shear, translation, rotation, or scaling.
	+ They are powerful tools used in CAD/CAM applications.
	− NURBS require more memory storage.
	− Improper initialization of weights can lead to bad parametrization.
Clothoids	+ Clothoids curvature changes linearly.
	+ Curvature continuity is easy to obtain.
	+ Clothoids can be used as transition curves in conjunction to other curves.
	+ Heavily used in railway track and highway road designs.
	− Fresnel’s integral might be difficult to compute.
	− Clothoid based planning uses global waypoints.
Interpolation Methods	+ Generally easy to compute.
	+ Curves can be concatenated to get desired shape.
	+ Fit for local planning for safety.
	− Difficult to control coefficients of curves of higher order (>4).
	− Curves of higher order are time consuming and not suitable for high speeds.
Hypocycloids	+ Easy to compute.
	+ Can be generated for desired angles.
	− $C^{2}$ continuity is not guaranteed.
	− Requires using transition curves (clothoids) for curvature continuity.
	− Not suitable for robots at high speeds.
Optimization Methods	+ Various constraints can be taken into account while optimizing.
	+ Can be combined with other approaches.
	− Depends on global pathways.
	− Optimization is time consuming and might not necessarily converge.
